# Quantifying the configurational complexity of biological systems in multivariate ‘complexity space’

**DOI:** 10.1098/rsif.2024.0558

**Published:** 2025-01-29

**Authors:** Tim Rock, Matthew A. Wills

**Affiliations:** ^1^ Milner Centre for Evolution, Department of Life Sciences, University of Bath, Bath BA2 7AZ, UK

**Keywords:** biological complexity, morphospace, information theory, macroevolution, morphology

## Abstract

An increasing number of evolutionary studies seek to quantify the morphological complexity of organisms, particularly those comprising serially homologous elements at different hierarchical levels of organization. Numerous operational frameworks have been proposed for doing this, but most focus on one or multiple conflated aspects of what is really a multidimensional concept. Here, we advocate the use of ‘complexity spaces’: multidimensional spaces defined by different vectors of complexity. We explore their application to biological systems composed of homologous parts and identify three axes on which those systems differ: part number, part differentiation and the regularity of that differentiation. Such complexity spaces can be constructed for systems at different hierarchical levels of biological organization. To illustrate this, we explore the complexity spaces for trilobite body plans (comprising body segments of varying number and form), and for ant colonies (comprising differentiated worker polymorphisms of varying number and form within a ‘superorganism’). Many different complexity spaces are possible, but all seek to distinguish different aspects of complexity within an information-theoretic framework, and thereby to clarify patterns of complexity evolution.

## 1. Introduction

Nature is characterized by nested systems made from multiple constituent parts [1]. Ecosystems comprise interconnected communities of organisms [2,3], and these organisms, in turn, comprise different organs and tissues [4]. Tissues comprise aggregations of individual cells [5], while cells comprise differentiated organelles and other molecular systems [6]. It is the proliferation and differentiation of these constituent parts at all hierarchical levels that produce biodiversity, and the aggregation of these constituent parts into successively more inclusive hierarchical levels (e.g. the ‘major transitions’ identified in [7]) that underpins the widespread notion that the maximum complexity of life has increased through geological time [8].

Surprisingly little attention has been given to circumscribing and defining the different aspects of complexity in biological systems. ‘Configurational complexity’, as usually conceived, encompasses numerous system descriptors which variously describe the organization of constituent parts at single or multiple hierarchical levels (i.e. systems can become more complex in multiple ways). Many of these organizational states evolve through different processes, and the configuration of parts at a given hierarchical level has knock-on functional effects upon the system [9]. Reductionist interpretations of this relationship tend to focus on the combinatorial potential of the interacting parts of a system [10,11], while holists and emergentists stress the bidirectional causality between levels in this hierarchical organization [12,13]. In either view, parts and wholes are interdependent [14], and their characteristics have important fitness consequences on one another [15]. As such, if we are to understand trends in biological complexity and the mechanisms that underpin its evolution through time, we must first develop a framework to quantify different forms of complexity with reference to this hierarchical structure.

While some complexity analyses have opted to focus on the geometric complexity of biological structures [16,17], a part-based (configurational) approach predominates in studies of complexity in deep time. For example, macroevolutionary changes in limb [18–20], vertebral [21–23] and body plan complexity [24] have been explored. These studies variously (albeit with little accordance) index complexity as some function of the number of parts, the extent to which those parts are differentiated from each other, and the regularity with which they are differentiated [25]. Hence, the ‘pure complexity’ (*sensu* [26]) of a system is established independent of any dynamic or functional system behaviours which arise from interactions between parts. While this approach is somewhat conservative, it is appropriate for quantifying complexity over macroevolutionary time scales, since interactions between system components are often poorly understood for fossil taxa [27,28]. It also provides indices that can be analysed relative to putative macroevolutionary drivers or constraints (e.g. ecology and climate).

Problematically, most empirical analyses of complexity utilize novel and ad hoc indices, depending upon the organisms or systems being studied. Such indices often combine and conflate multiple aspects of complexity into a single number [20,21]. This ignores important differences between distinct types of configurational complexity, obscuring an understanding of both system structure and the processes that formed those structures [29]. To illustrate this issue, consider the contrasting patterns of evolution in the skeletal system versus the brain of mammals. Mammalian evolution is characterized by the differentiation of homologous skeletomuscular elements within the same body plan [30]. While the numbers of some of these elements are variable (e.g. vertebrae within the post-cervical spine), it is predominantly the morphological differentiation of elements relative to each other that yields greater morphological complexity within organisms [21,31]. Function and constraint in this context are contingent almost entirely upon part differentiation, with losses and gains of parts being less common. In contrast, all tetrapod brains comprise the same basic components (e.g. neurons, glial cells, capillaries) [32–34], but vary hugely in the number and connectivity of these components in different regions (e.g. shrew brains have approximately 9.7 × 10^6^ neurons, while humans have approximately 1.6 × 10^10^) [35]. Brain mass therefore varies by over three orders of magnitude, correlating with vastly different levels of cognitive ability and associated metabolic costs [36]. No single index of complexity could faithfully capture the multivariate nature of complexity in these examples. This is not to deny the utility of single or composite indices for certain applications [18,20,37], rather to highlight the need for a multidimensional approach if the dynamics and drivers of complexity change are to be more fully understood.

Beyond the physical structure of systems alone, the multivariate nature of complexity emerges in the distinction between putative mechanisms that drive complexity change. For example, consider the zero force evolutionary law (ZFEL [38]) and the theory of constructive neutral evolution (CNE [39]). Both the ZFEL and CNE are mechanisms claimed to cause passive increases in aspects of complexity through time. However, while the ZFEL predicts that serial homologues will passively differentiate in the absence of selection to the contrary (increasing complexity along the ‘differentiation’ axis), CNE predicts the retention of non-functional parts, leading to an increase in part number through time (increase along the ‘element number’ axis). Similarly, others have argued for the selective importance of division of labour [40,41], putatively driving system complexity through the enhanced energy efficiency of parts specialized for specific functions. However, the relative importance of increased part differentiation or increased part number in achieving this objective is unclear. In order to address such issues, we stress the need for an operational framework that (i) accounts for the multivariate nature of configurational complexity change, and (ii) provides a set of universalizable indices which can be applied to biological systems at all levels of hierarchical organization.

**Figure 1.**
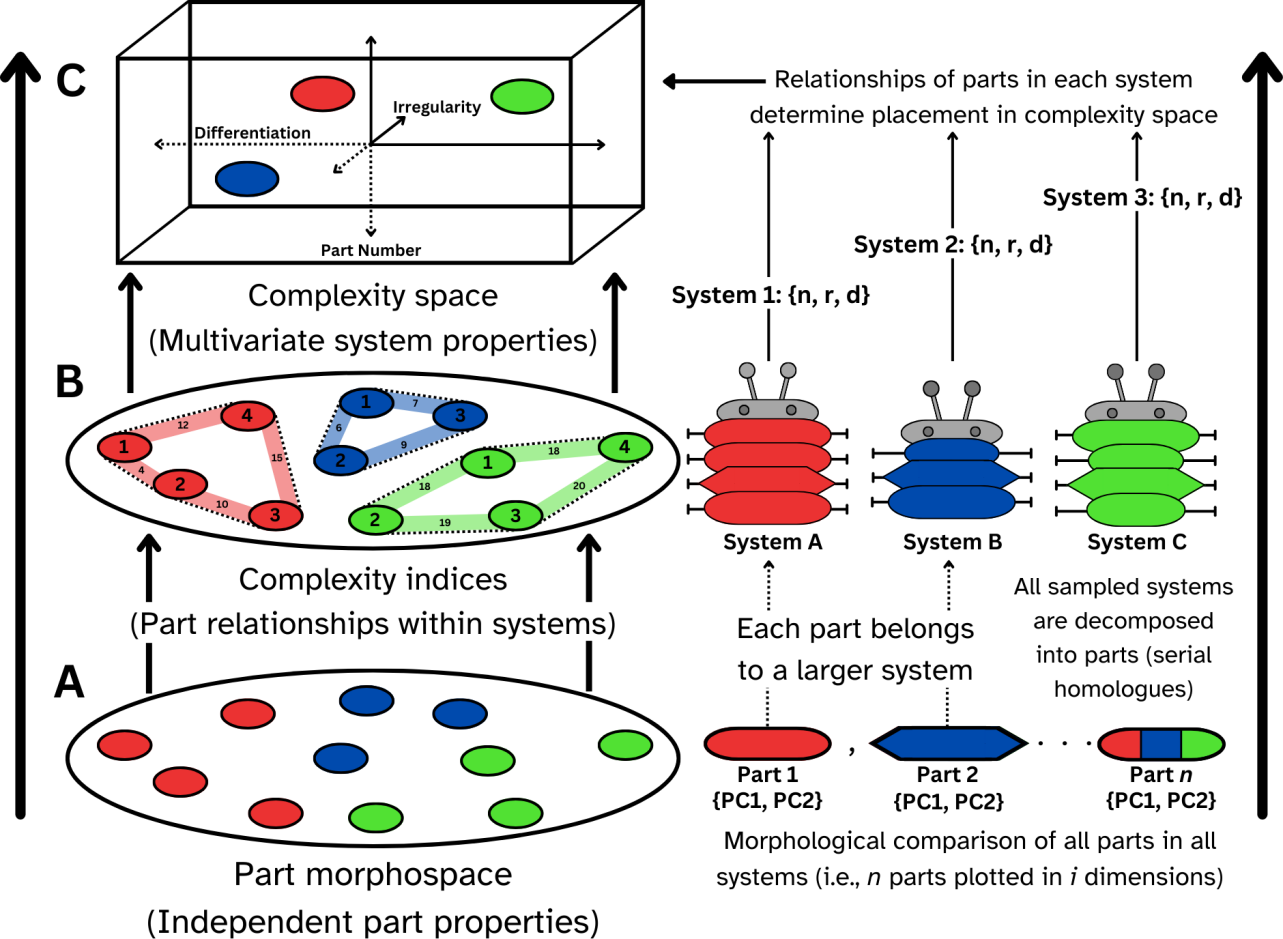
A comparative analysis of system complexity can be made in complexity space, aspects of which can be derived from a morphospace. This begins with (A) performing a morphometric analysis of every serially homologous part (e.g. limb/body segment) in a sample (irrespective of the system each part belongs to); (B) indices of system complexity are then derived from the relationships between the constituent parts of each system within the morphospace (e.g. number of parts and their degree of differentiation); and (C) systems are plotted relative to new axes defined by the complexity indices (illustrated as variables *n*, *r*, *d* calculated in Step (*b*) (and/or derived from elsewhere).

To this end, we advocate ‘complexity spaces’ as multidimensional tools for visualizing the complexity of systems comprising duplicated and differentiated serial homologues. In general, we propose spaces defined by three axes of complexity: number of parts, differentiation of parts and the regularity of the arrangement of those parts (figure 1). This draws heavily from McShea [25] and Fusco & Minelli [24]; however, we generalize their approaches to measure systems that are non-sequential (i.e. where parts do not have an obvious order/sequence as they do in a vertebral column or a series of limbs). We believe that this framework has broad application across disparate biological systems and levels of organization.

We draw parallels with attempts to index the morphological disparity of groups of species within clades or time bins [42–44], whereby there is no single disparity index that captures all aspects of morphological diversity [45]. A fuller picture of disparity and disparity change therefore necessitates the use of multiple indices, and in particular an exploration of how these indices interact [46]. Similarly, there is no single, universally applicable index of configurational complexity and we therefore advocate a plurality of indices wherever practicable.

## 2. Building a complexity space

Morphological disparity (as opposed to complexity) indices are used to quantify the relative morphological variety in different groups of species (or organisms sampled from those species) [47]. These species are typically partitioned into time bins (longitudinal studies of disparity through time [48]) or clades (comparative studies across trees [46]), but other subdivisions are possible (e.g. into ecological guilds or habitats [49]). Each species is described by a number (*n*) of variables. These can be geometric morphometric variables, continuous measurements, discrete characters or some combination of these [43]. All species are plotted with respect to all variables in an *n*-dimensional hyperspace, and aspects of the distributions of groups of species within this hyperspace (e.g. sum of variances (SoV) on all axes or mean pairwise distance) constitute several possible indices of disparity [43,50,51].

In the same manner that morphospaces can be used to realize the distributions of species within clades or other groups, morphospaces can be used to visualize the distributions of serially homologous elements within biological systems. The limbs of arthropods, for example, might be described by a set of *n* variables, all of which can be coded for all serial homologues down the body of an individual, but also for limbs across species or higher taxa. By plotting all limbs from all species within a single morphospace, limbs rather than organisms or species become the points (figure 1). Organisms are then represented by a scatter of points, whereby those with closely similar limbs have tighter clustering than those with strongly differentiated limbs. Some pre-existing disparity indices are then applicable to quantifying complexity on the ‘differentiation’ axis of a complexity space. For example, the SoV of groups within morphospace is a commonly used disparity index [50], summarizing the variation of operational taxonomic units along all included axes. It has the twin advantages of insensitivity to the orientation of the reference axes, and relative insensitivity to sample size differences (e.g. the number of species or higher taxa). Analogous indices can be used to quantify how differentiated the parts of systems are relative to each other. This builds upon McShea and Brandon’s [38, p. 10] recognition that ‘[disparity] at system level *N* is just its complexity at level *n +* 1’. Inevitably, indices of complexity—like their disparity analogues—are relative, not absolute, and contingent entirely on the framework of variables used for their quantification [52]. Nonetheless, they can be used to identify patterns and trends in complexity across groups and through time, and as covariates in analyses of macroevolutionary patterns.

Multiple, independent complexity indices can be used to define the orthogonal axes of an abstracted complexity space or hyperspace. Here, we illustrate three-dimensional complexity spaces (derived from three complexity indices) but note that complexity spaces can be defined by any number of indices, provided that these are logically and empirically decoupled. Although complexity spaces are abstractions from indices that may themselves be derived from morphospaces, they represent variations in types of configurational complexity rather than variations in form. Systems (e.g. groups of organisms) plotted in complexity space can be understood not just as *more* or *less* complex than one another, but also as *differently* complex.

## 3. Quantifying complexity using information theory

All of the complexity indices used to construct complexity space can be considered in terms of the amount of information necessary to describe various aspects of system structure. We follow others who have developed measures of system complexity based on a theoretical encoding–decoding process where system configurations are expressed as ‘messages’ in as few characters as possible while maintaining the potential for lossless decompression [52,53]. In this framework, part configurations that can be described in fewer characters are considered less complex than those which require a greater number. Central to this approach are the concepts of compression and redundancy, whereby repetitive or similar values in a distribution can be summarized in fewer characters.

To illustrate this, consider two sequences,


Sequence 1: {A, B, A, B, A, B, A, B, C, D}

Sequence 2: {A, B, D, C, B, D, A, A, B, D}.

Although both sequences comprise 10 of the same four characters (A, B, C, D), Sequence 1 can be considered less complex because it exhibits regularity (a repeating pattern of ‘A, B’ for most of its length). Sequence 1 can be compressed from 10 characters to seven without losing any of the information in the original description as


$$Sequence\;1:\;\left\{{\left(AB\right)}^{4},\;C,\;D\right\}.$$

However, this is not true of Sequence 2, as there are no aspects of the configuration that allow a reduction in the number of characters used. Sequence 1 exhibits redundancy while Sequence 2 does not. This same logic can be applied to morphological data used to describe the distribution of the parts of a system within a morphospace. For example, Sequences 1 and 2 might be numerical values describing the width of each somite down the bodies of two arthropods, or vertebrae down the spines of two mammals. In either case, the organism represented by the first sequence would be considered less complex than that represented by the second.

Several studies have applied similar approaches to quantifying the complexity of both biological and non-biological systems [52,54,55]. While these approaches recognize that the number of parts in a system does not linearly increase the information required to describe it (and thus its complexity), they have all sought to express information content in a single number or sequence. Unfortunately, this conflates different aspects of configurational complexity, and the length of descriptions is contingent on the chosen encoding process, since different forms of information can be compressed better under some regimes than others [56]. This adds another layer of abstraction that will inhibit the comparison of complexity across groups.

Instead, we establish various indices of configurational complexity based upon aspects of system structure that determine the Shannon entropy of parts within system descriptions. Entropy indicates the uncertainty of a variable occurring in a distribution [57] and can be applied to systems to describe the uncertainty of multiple variables. Low-entropy outcomes tend to require less information to describe than high-entropy ones [58]. Thus, entropy and description length depend on the range and probability of values. For example, a coin toss has lower entropy than a six-sided dice roll, and so describing the outcomes of multiple tosses will tend to require fewer characters to describe than multiple rolls. System entropy scales with the number of outcomes, the distribution of possible values and the independence of successive outcomes. Fewer outcomes, smaller ranges and less independence reduce entropy and description length.

This framework can be extended to analyse the distribution of points in a system across morphospace, based on the morphology of the parts of each system. In Shannon’s [59] communication example, the entropy of a message is calculated by the number of characters relative to the number of possible values each character can have (e.g. the 26 letters of the Latin alphabet). Similarly, in morphospace, systems have a variable number of parts assigned values along multiple axes based on their morphology. The range of possible values is consistent across systems within a given morphospace. While entropy does not provide an exact measurement of information content, it defines the minimum possible length of a description [58]. Here, we consider three forms of complexity commonly indexed in macroevolutionary analyses. For each, we discuss how they can be operationalized in morphospaces relative to the concept of information and Shannon entropy.

## 4. Number of parts

Given the above framework, we define systems with more parts as more complex than those with fewer parts. Adding parts can only increase or maintain the description length and expands the combinatorial space of possible outcomes, thus increasing uncertainty [57]. Here, parts refer to homologous structures identifiable across a sample of systems at a given hierarchical level. Provided that each system can be broken down into homologous parts, this approach is extendable to various organizational levels, such as comparing organ complexity within organisms or organism complexity within ecosystems.

## 5. Degree of part differentiation

The second index of complexity applied here is the degree to which the parts of a system are differentiated. Systems with parts that are more differentiated from one another are considered more complex than those that are less differentiated. Differentiation can be indexed using several indices of spread on multiple dimensions of a morphospace, including the sum of univariate variances, SoR and mean pairwise distance [43,44]. These are more familiar in their application as indices of the disparity of groups of species in time bins, clades or other groups.

Morphospaces can be derived from discrete variables (e.g. presence/absence characters and other discrete ‘cladistic’ characters including counts and meristic variables), from continuous variables (such as linear dimensions and subtended angles), from geometric morphometrics (e.g. landmarks and semi-landmarks), from outline descriptions (e.g. Fourier transforms and eigenshape analyses) or from combinations of such data. Discrete character morphospaces have discrete probability distributions along each axis, like those for coins or dice, albeit that each state need not be equiprobable. For other types of morphospace, values are distributed along continuous axes, and so the probability of any given value occurring is vanishingly small. Hence, empirically observed values in non-discrete morphospaces must be considered relative to a probability density function [56]. Systems whose parts are more dispersed along a given axis will tend to require a greater amount of information to describe to a given degree of precision than systems where parts are less dispersed. Where the dispersion of a distribution is greater, the uncertainty of predicting a given value increases. Furthermore, if we were to discretize a continuous morphometric axis and assign each point into a bin, then the most differentiated systems would tend to fall into the greatest number of bins (and thus require more characters to describe) as the resolution of this scale increased and the size of each bin approached zero [56]. Hence, systems with parts that are differentiated to a greater degree will tend to require longer descriptions than those with parts which are differentiated to a lesser degree.

## 6. (Ir)regularity of part arrangement

Our third index of complexity is the regularity with which parts are morphologically differentiated. Regularity is measured independently from the degree of part differentiation, such that the same index of regularity could be reported for systems with orders of magnitude difference in part differentiation. Similarly, parts may be highly differentiated in a very regular way, or much less differentiated in an irregular way.

On a single, discrete axis of differentiation, irregular systems are understood as more complex because a greater number of values is needed to describe the distribution of points along the axis. Along continuous axes, a probability density function can be applied (as used in degree of differentiation). Where the distances between the parts in a system have low variance, the distribution can be understood as less complex. For example, in a system where *n* parts are approximately (but not precisely) equally spaced along a continuous one-dimensional axis, their placement will be easier to approximate than the *n* parts of a system distributed irregularly or scattered randomly. An irregular distribution entails a higher degree of uncertainty and greater entropy, and therefore more information is needed to describe it.

Unfortunately, quantifying the irregularity of a point distribution (here, the distribution of parts in an *n*-dimensional morphospace with continuous axes) at all spatial scales simultaneously is a notoriously difficult problem. Based upon the information-theoretic framework outlined above, a perfectly regular distribution would be one in which all parts were equidistant from one another. This would mean that only one value would be required to encode all of the distances between all of the parts. This is possible for three points in two dimensions (points on the vertices of an equilateral triangle or 2-simplex) and four points in three dimensions (vertices of a regular tetrahedron or 3-simplex). In general, *n* points can be placed symmetrically in *n* − 1 dimensions so that each point is at the same distance from every other point. Given this, operationalizing our definition of regularity is dependent on both the number of parts a system has, and the number of dimensions which define the morphospace.

When systems in a sampled group comprise a relatively small numbers of elements and are ordinated in a space of high dimensionality, it is perfectly possible for all elements within a system to be equidistant from all others. Therefore, where the dimensionality of a morphospace is one less than, equals or exceeds the number of elements being considered, regularity can be legitimately indexed as the variability of all possible neighbour distances at all orders. More precisely, the coefficient of variation (CV) or relative standard deviation (equivalent to the standard deviation divided by the mean) of all pairwise distances between elements offers an index of regularity that is independent of the magnitude of the distances themselves (e.g. the mean pairwise distance or the sum of univariate variances). Systems with a lower CV are deemed less complex than those with higher CV on this axis, with perfect regularity corresponding to CV = 0.

In contrast, when systems in a sampled group have a greater number of parts than the number of dimensions which define the morphospace, it is impossible for all elements belonging to these systems to be equidistant. In a regular, square two-dimensional lattice, the first- and second-order nearest neighbour distances (NNDs) will all be identical. However, higher orders of NNDs will be greater, and the variance of all possible neighbour distances will be non-zero. Given this constraint, and as a pragmatic solution, we use the CV for all first- and second-order NNDs as our index of regularity. However, we note that while the CV of lower-order NNDs captures regularity at local scales it is insensitive to the reduced entropy of clustering at higher spatial scales. The most suitable index is therefore contingent upon both the number of parts and the dimensionality of the morphospace.

We acknowledge that many more sophisticated indices of spatial regularity are possible [60,61]. To the extent that they can be applied in *n* dimensions and are independent of the degree of differentiation, all could be deployed as alternatives and refinements for particular applications. To this end McShea [25] and Fusco & Minelli [24] devised indices of the regularity of differentiation between adjacent serial homologues (e.g. vertebrae within the vertebral column and somites within the arthropod body). However, their application requires that there is a known or assumed spatial, ontogenetic or evolutionary sequence of homologues within a system. While capturing similar patterns as Fusco & Minelli’s [24] ‘Cs’ index of regularity (Appendix A), indices such as the CV may have wider utility, may make fewer assumptions and can capture patterns other than those occurring in serially adjacent homologues.

## 7. Examples of complexity spaces

We offer examples of applying the complexity space concept to biological systems at two different levels of organization. First, we measure the configurational complexity of trilobite body plans that vary in the number and overall dimensions of thoracic segments. Secondly, we measure the configurational complexity of Attine ant taxa, varying in the number and form of worker polymorphisms. These examples are offered as proofs of concept, rather than being intended to sample either clade comprehensively or to address particular macroevolutionary hypotheses. This is especially true in our trilobite example, where we derive part measurements from idealized, illustrated reconstructions, rather than articulated fossils or photographs of those specimens. While we offer some interpretation of the results of our analyses for illustrative purposes, we recognize the limitations of these data and eschew firm conclusions, deferring to those who work on both groups.

## 8. Trilobite segment complexity

The bodies of arthropods are composed of serially homologous somites and limbs. These have proliferated and differentiated throughout their evolution [62,63], although there are numerous examples of somites being lost [64,65] or fused [66] and of limbs being lost [67]. These processes have produced enormous variety in the morphology and configurational complexity of arthropods. They also underpin the process of tagmosis, whereby segments become integrated to form functional units [68].

Excellent studies have focused on singular aspects of trilobite complexity such as the number of segments in different body regions [42,69,70] or have analysed disparity through time and across clades [71,72]. Here, we characterize the segmental complexity of a taxonomically broad but otherwise limited sample of trilobites on three axes of configurational complexity: number of elements, differentiation of elements and regularity of differentiation. For simplicity and illustrative purposes, we focused on the thorax, although we note that disparity analyses have also restricted themselves to particular regions such as the cranidium [73] or cephalon [74].

**Figure 2.**
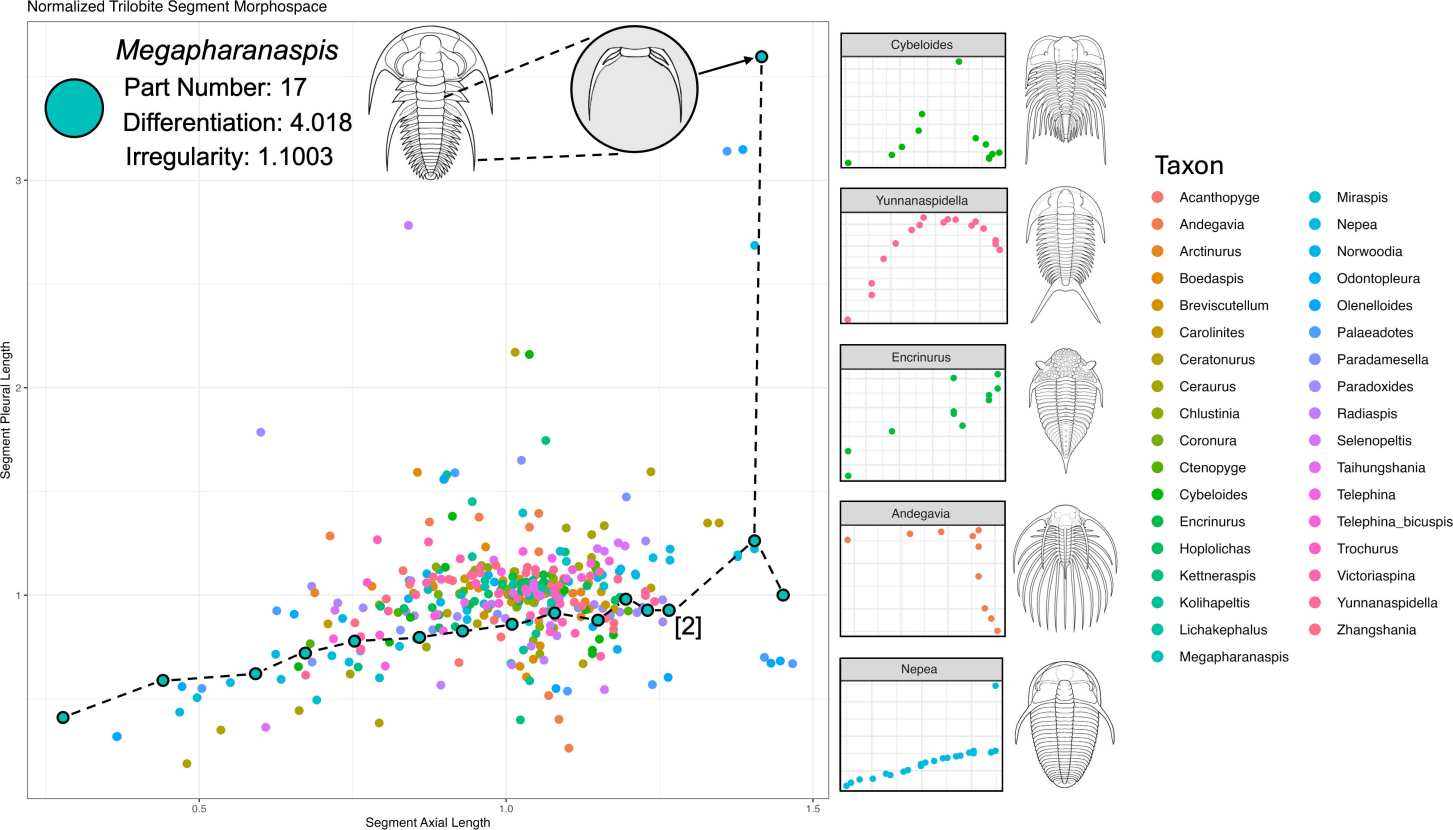
Morphospace of trilobite segments, constructed from measurements of the mediolateral length of the axial and pleural lobes of each thoracic segment. The segments of one trilobite, *Megapharanaspis*, are highlighted with black circles, and joined by a minimum spanning tree [76] for illustrative purposes only. In *Megapharanaspis*, the segments appear to ‘progress’ from posterior to anterior along the *X*-axis. However, the morphospace does not explicitly order the segments in this way, and it is largely contingent that the axial segment lengths increase in this way. The complexity indices calculated for *Megapharanaspis* from this morphospace are also shown. Note that only 16 points are visible, with the superimposed locations of two segments being indicated by [2]. Axes of segment distributions shown for other trilobites (pictured right) are scaled to their respective ranges for clarity, not the actual ranges of their distributions in morphospace. Independent distributions for all taxa are shown in Appendix D (Figure 8). Trilobite line illustrations reproduced from Samuel M. 'Ohukani'ōhi'a Gon III.

The number of thoracic somites (axis one of the complexity space) entailed a simple count. To obtain scores on the ‘element differentiation’ and ‘element regularity’ axes (axes 2 and 3), we first produced a simple morphospace of all somites from all our exemplary trilobites. Such a morphospace could have been derived from an array of discrete (‘cladistic’) characters [75], geometric morphometric or outline data. Here, we used just two linear measurements to define a two-dimensional morphospace: namely, the mediolateral lengths of pleural and axial lobes (figure 2). Measurements were performed on 36 genus-level reconstructions (electronic supplementary material, supplementary information) from a dorsal perspective using ImageJ (v. 1.54 [77]), producing measurements for a total of 385 individual segments. To control for differences in size across species, each lobe measurement was divided by the mean of all measurements for the respective lobe type in each trilobite.

Degree of part differentiation for each trilobite was measured as the median SoV of all segments of each taxon in this morphospace, bootstrapped with 100 pseudoreplicates to a sample size matching the number of segments for each taxon, respectively. This was performed using the ‘DispRity.per.group’ function in the *DispRity* (v. 1.8 [78]) package for the statistical programming language R (v. 4.2.1 [79]).

In the context of indexing disparity (and, by extension, this axis of complexity) a plurality of approaches is often recommended [43,44]. In particular, the sum of ranges (SoR) is often used as an index that captures the maximum bounds of variation. However, SoR has some undesirable properties, not least that it is contingent on the axes against which it is measured. If a cloud of points is rotated at random (while maintaining the spatial relationships between those points), then the SoR is likely to change, while the SoV will not. The use of SoR would be particularly problematic in our trilobite example, where we refer taxa to our two original variable axes rather than to the axes of an ordination. One solution to this arbitrariness is to rotate the cloud on principal component axes using the covariance structure of the data. However, this is a variance-based approach, and it is not obvious why one would subsequently discard this logic to look at ranges. Another undesirable property of the SoR is its greater sensitivity to sample size differences. Rarefaction and other approaches can control this, but the SoV is much less susceptible from the outset. Regularity of part arrangement was calculated using the CV of first- and second-order NNDs of each segment of each trilobite in morphospace.

## 9. Results and discussion: trilobite segment complexity

Our trilobite complexity space demonstrates that genera realize their complexity in a variety of ways, and that configurational complexity can change along independent axes through time. Moreover, our modest dataset demonstrates that multivariate complexity change can be understood with a few, simple measurements. For example, *Radiaspis* has relatively few thoracic segments, but these are differentiated in a highly irregular pattern (Appendix B, table 1). In contrast, *Coronura* has many more segments; however, these are differentiated more regularly. Similarly, *Nepea* has more segments than its near-contemporary *Megapharanaspis*; however, *Nepea* shows greater irregularity, while *Megapharanaspis* shows a greater degree of differentiation.

**Figure 3.**
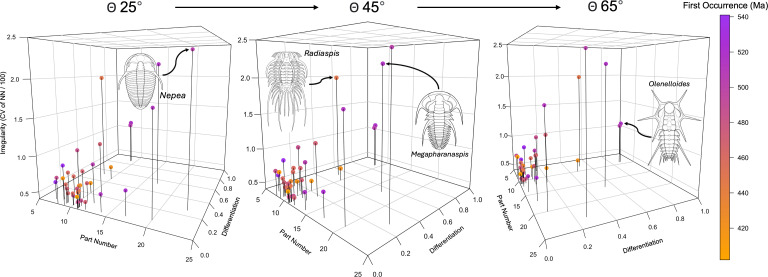
Complexity space of trilobites at different (arbitrary) angles to the viewer. First occurrences of genera are indicated by colour coding, from oldest (purple) to youngest (orange). Our sample shows a tendency for reducing configurational complexity on all axes through time. Trilobite line illustrations reproduced from Samuel M. 'Ohukani'ōhi'a Gon III.

**Figure 4.**
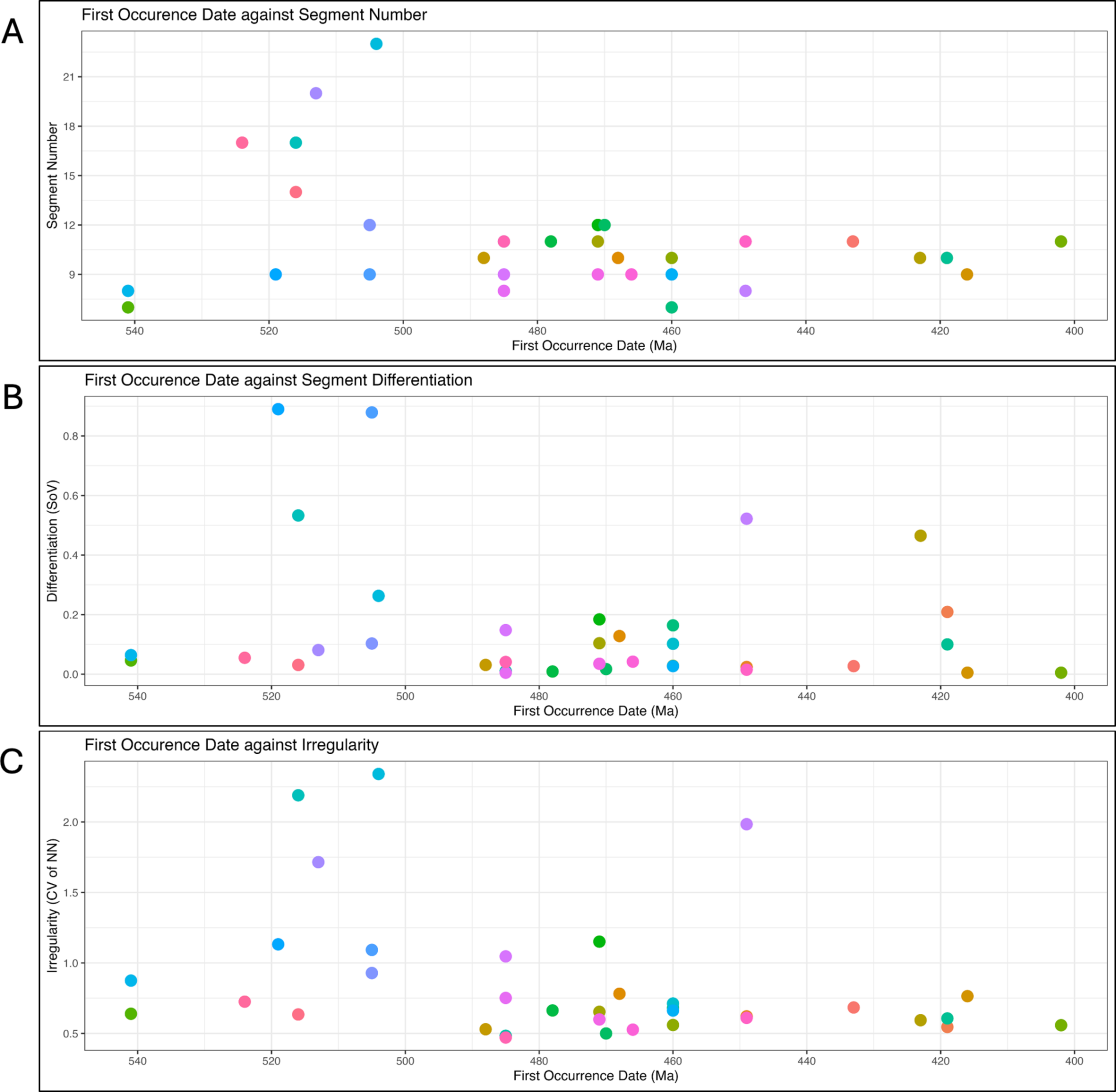
Changes in independent complexity indices for trilobite genera through time. Taxa are coloured according to genus, consistent with the colouring in figure 2. (A) Thoracic somite number against first occurrence date. (B) Degree of segment differentiation against first occurrence date. (C) Irregularity of differentiation against first occurrence date.

Older taxa tend to have higher complexity on all three axes than younger taxa (figures 3 and 4). The Cambrian genera *Megapharanaspis* (516 Ma), *Olenelloides* (519 Ma) and *Palaeodotes* (505 Ma) have the greatest thoracic somite differentiation in our sample, while the Cambrian genera *Nepea* (504 Ma)*, Paradoxides* (513 Ma)*, Yunnananspidella* (524 Ma)*, Megapharanaspis* (516 Ma) and Z*hangshania* (516 Ma) have the greatest number of thoracic somites. At the same time, *Nepea, Megapharanaspis* and *Paradoxides* exhibit the first, second and fourth highest degree of irregularity. By comparison, the most recent taxon, *Coronura* (402 Ma) has only 11 thoracic segments, which are the least differentiated in the whole sample (equal to *Taihungshania* and *Breviscutellum*) and are differentiated in a regular manner.

This pattern is broadly consistent with prior work focused on single indices of complexity. Hopkins & To [69] noted a decrease in the maximum number of thoracic segments in trilobite species from the Cambrian to the Permian. This was coincident with convergence towards the median number of segments (twelve in the Cambrian and nine in the Permian) over the same period. While our sample of trilobites is much smaller and not phylogenetically representative, it illustrates examples of extremes of configurational complexity that reduce through time on multiple axes. This pattern of reducing complexity might seem counter to expectations. Both passive diffusion and the ZFEL [38] predict that somites would become less similar and more irregular in the absence of selection acting to the contrary. This would result in an increase in complexity on two of our three axes (differentiation and regularity).

Heterochrony has been extensively implicated in the evolution of trilobites [80], with both peramorphic [81] and paedomorphic [82,83] trends being proposed. After the Cambrian, many trilobite groups independently evolved planktonic larvae [84], developing conspicuous spines from the cephalon and pleurae to afford some protection and slow their rate of descent [85]. In several lineages, these spines were ultimately retained in the much larger adults, constituting convergent paedomorphic shifts in groups including Odontopleurida [80], Encrinurinae [86], other Lichida [87] and Trinucleids [88]. To the extent that this resulted in greater differentiation and irregularity of pleural shapes through time, this might have been expected to result in increasing complexity indexed on both the differentiation and regularity axes.

While recognizing again that our dataset is limited in sample size and contains measurements taken from idealized reconstructions, our results therefore suggest the possibility of selection or clade sorting [89] acting in opposition to reduce complexity on these two dimensions. In contrast, mitotic heterochrony [90] could result in changes to the number of body somites, which have tended to decrease during the course of trilobite evolution [69,91]. This is also a paedomorphocline, but one that would tend to reduce complexity on the part number axis. The relatively high complexity of Cambrian trilobites is nonetheless counterintuitive. Certainly, there appear to have been fewer developmental constraints on somite numbers across Cambrian trilobite species relative to their post-Cambrian descendants. Moreover, Cambrian species also showed greater variation in intraspecific somite numbers than those from later strata [92,93]. Looser ecological constraints in the Cambrian may have facilitated more rapid evolution of novelties [94–96], while weaker pleiotropy in developmental programmes may have resulted in less constrained body plans [97]. In other contexts, Brinkworth *et al.* [19] suggested that more complex body plans may limit morphological diversity and species richness because they also tend to be developmentally entrenched. As parts increase in number and differentiate, the stability and integration of a system increases at the cost of adaptability [98].

## 10. Ant polyphenism complexity

Eusocial insect colonies are examples of adaptive units that extend beyond their individual constituent organisms [99]. In ants, colonies are often regarded as ‘superorganisms’ given their haplodiploid breeding system whereby a sterile worker caste enables (fertile) queens to reproduce *en masse* [100]. Workers produced by the same queen have a coefficient of relatedness of 0.75 with each other, such that by enabling the queen to produce more of their full siblings, workers increase their own fitness [101,102]. Whole ant colonies composed of thousands of individuals can therefore be viewed as integrated reproductive units [103].

Alongside the reproductive division of labour, workers often perform unique roles such as food procurement, colony defence, brood care and nest maintenance [104,105]. In many species, these roles are performed by conspecifics that are behaviourally differentiated. However, many taxa also exhibit a morphological expansion of the worker caste to include major and supermajor workers (constituting dimorphic and trimorphic worker polymorphisms, respectively) [106]. Analogous to the multiple differentiated homologous parts of single organisms that perform unique roles, morphologically differentiated workers are specialized for different tasks necessary for colonial success [107,108]. While this theoretically provides fitness benefits through heightened efficiency in the division of labour, it also means that workers are more integrated, and so a given worker may be less suited to a generalist set of behaviours [109]. Notably, however, the determination of an individual worker’s polymorphism occurs at the embryonic stage and is regulated in part by environmental and demographic cues [104]. As such, the proportions of morphologically differentiated workers within a colony are not fixed throughout the lifetime of a single queen, and so the relative abundance of certain part types within this system can change as necessary (unlike single organisms that mostly exhibit the same morphology after sexual maturity).

The social and morphological ‘complexity’ of ant taxa has been investigated before, but a wide variety of methods and approaches have been used. As with studies of the morphological complexity of single organisms, analyses often quantify complexity using novel and ad hoc indices that either focus on single aspects of system complexity or conflate multiple aspects into a single score [110,111]. Here, we focus strictly on distinct indices of phenotypic configurational complexity as axes in our complexity space but include the minimum colony size of each species (gathered from an AntWiki referenced database) as a fourth variable to be considered relative to complexity space occupation.

To explore the relationship between division of labour and morphological complexity in attine ants (Myrmicinae; Attini), we quantified variation in the two-dimensional outline of frontal views of the heads of 64 species using elliptical Fourier analysis (EFA). Photographs of queens, males and workers were garnered from AntWeb [112], an online database validated as a resource for accurate morphometric analyses of body segments [113]. Attine ants were chosen because they exhibit monomorphic, dimorphic and trimorphic caste polymorphisms. This enables comparison between species with differing numbers of worker forms (elements). Additionally, attine ant colonies are predominantly monogynous (containing a single queen) [114], and so colonies can reliably be viewed as a single reproductive unit.

Previous research on the disparity of worker castes in ants has focused on the size variation of subcastes [115]. Here, we controlled for differences in size to focus on intraspecific variation in head shape. Our dataset differs from most others used to quantify variation within species [116,117] in that we include all polyphenisms (queen, male and all workers) rather than only comparing worker polymorphisms, all of which can also vary significantly within a single taxon.

We note that the designation of discrete castes is somewhat subjective in many species. However, our analyses are primarily intended as proofs of concept rather than as definitive explorations of these aspects of ant evolution. As a sampling methodology, monomorphic species were chosen (where the head shape of workers changes isometrically with increased size), and dimorphic workers (majors and minors) were sampled according to delineations on AntWeb. Trimorphic taxa (all of the genus *Pheidole*) were sampled according to Wilson [118]. Additionally, taxa were only included in analyses if all photographs for all polyphenisms were available (male, queen and all worker polymorphisms) on AntWeb.

**Figure 5.**
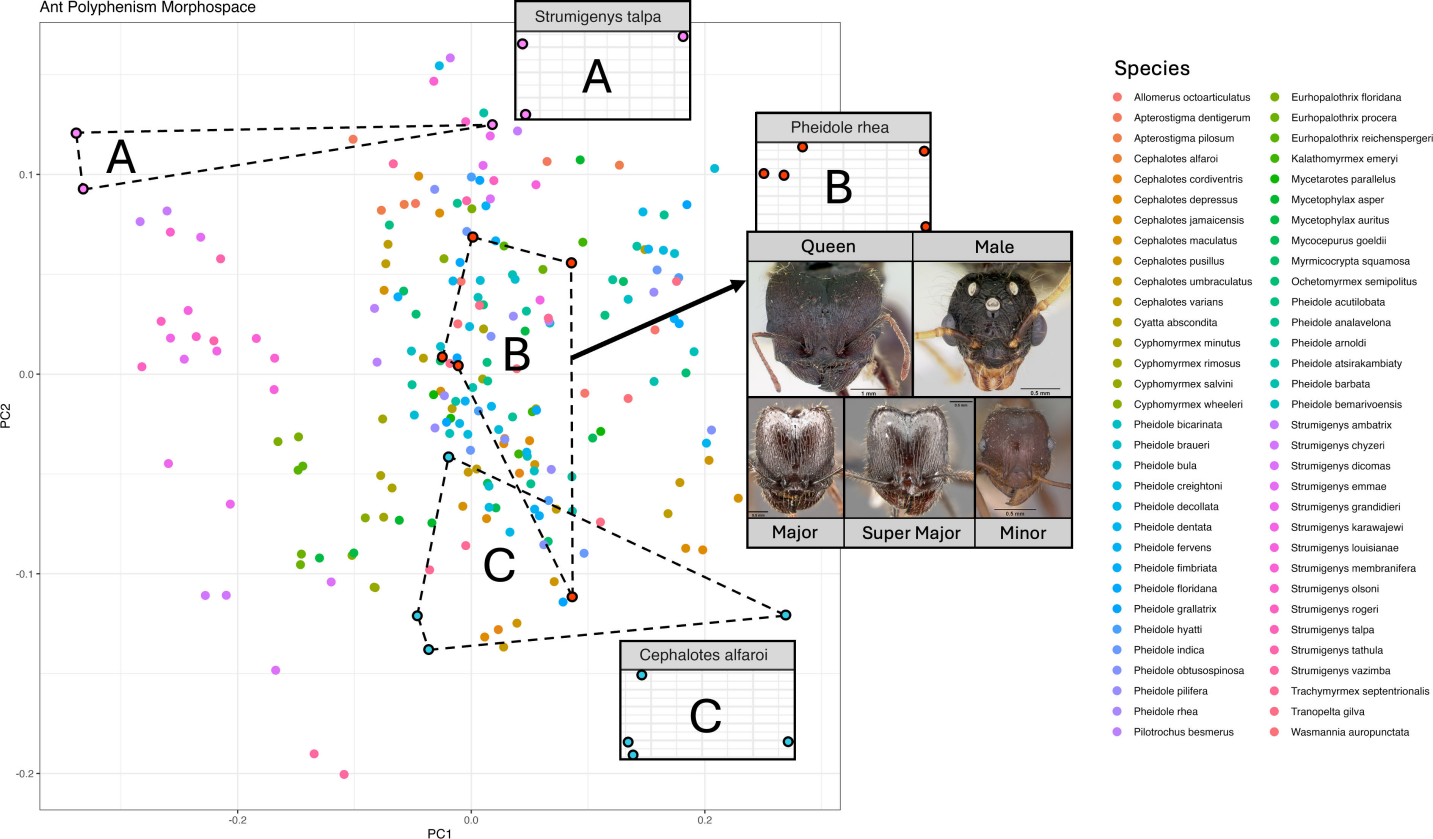
First and second principal component axes of a morphospace of attine ant polyphenisms, constructed from outlines of heads captured using EFA. Points of the same colour are polyphenisms of the same species. Inset photographs are those used to quantify polyphenisms for *Pheidole rhea*, a species with trimorphic caste polymorphism. Axes of distributions shown for individual ant species are scaled to their respective ranges. Independent distributions for all taxa are shown in Appendix E (Figure 9). Photos taken from AntWeb.

Outlines of each head were generated as binary masks from AntWeb photos taken from an anterior viewpoint in ImageJ [77]. Overall, 64 species were sampled, comprising 225 individual polyphenisms. Procrustes transformation was performed to control for size, position and orientation using the R package momocs (v. 1.4.1 [119]). Head shape was quantified using EFA and all outlines were ordinated using principal component analysis (PCA) (figure 5).

The number of parts in each species was counted as the number of polyphenisms (male, queen and worker polymorphisms). Monomorphic species therefore have three ‘parts’, while dimorphic and trimorphic species have four and five, respectively. As with our Trilobite dataset, the degree of part differentiation was calculated using the bootstrapped SoV. This utilized 100 pseudoreplicates, each with a sample size equivalent to the number of polyphenisms in each species. We performed these calculations for all polyphenisms of a species within the PCA, utilizing the DispRity package (v. 1.8 [78]), for the R statistical programming language (v. 4.2.1 [79]). Finally, the regularity of differentiation was calculated as the CV of first- and second-order NNDs of all polyphenisms of each species.

## 11. Results and discussion: ant polyphenism complexity

Social insects offer excellent model systems for exploring the relationships between morphological diversity, modularity and integration, as well as the division of labour in biological systems. Several prior studies have quantified the number and degree of caste polymorphisms as indices of complexity [120–122]. Others have focused on size and shape differences between distinct worker polymorphisms [116,117,123,124]. Here, we quantify the nature and magnitude of shape differences between queens and males, as well as between worker polymorphisms, and use these data to calculate the complexity of species on our ‘part number’, ‘differentiation’ and ‘regularity’ axes.

Multidimensional approaches to measuring complexity in social insects are not new. Anderson & McShea [106] distinguished four domains of social complexity: polyphenism, individual totipotency, work organization and communication. More recently, Holland & Bloch [125] showed that social complexity in insects increases nonlinearly when complexity is delineated into multiple indices, necessitating a multivariate approach, as we advocate here. However, while they highlight many variables of interest (e.g. queen–worker dimorphism and worker variation), they ultimately conflate these indices into a ‘combined social complexity index’ alongside non-morphological indices such as colony size and longevity. Here, we stress the value of distinguishing between social and morphological complexity, and then once again between specific types of morphological complexity, which can be considered independently. Against this ‘pure’ configurational complexity [26] framework, there is potential to investigate the correlation between morphological complexity and indices of social complexity (e.g. colony size, cooperation and behavioural specialization).

**Figure 6.**
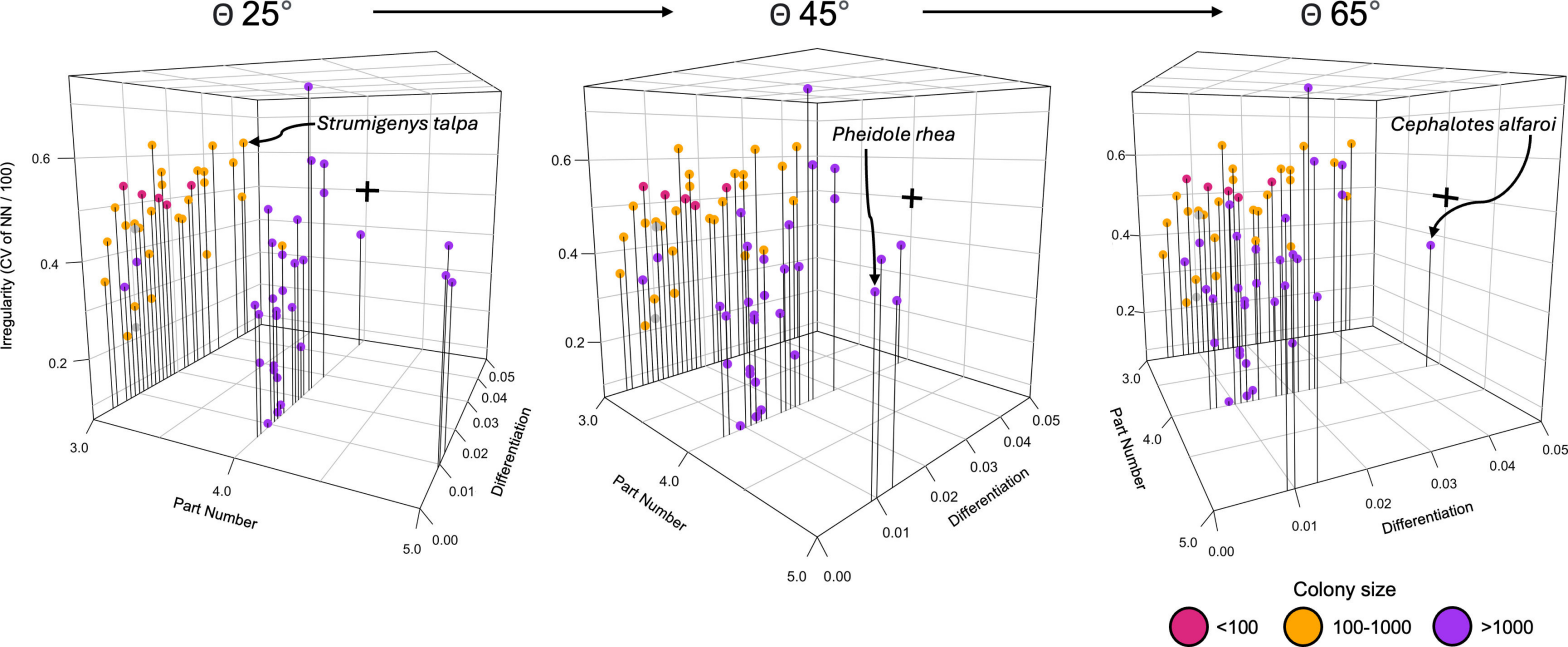
Complexity space of attine ant colonies constructed using EFA of two-dimensional head outlines. Part number appears to strongly correlate with colony size, while other complexity indices show no correlation. Monomorphic species live in smaller colonies, while dimorphic and trimorphic species live in larger colonies.

Our complexity space (figure 6; Appendix C; table 2) demonstrates that species can vary in complexity independently along all axes. Although the lower bound of head differentiation increased in species with a greater number of worker polymorphisms, monomorphic species exhibited a higher upper bound of morphological differentiation than all dimorphic species except *Cephalotes alfaroi* (Appendix C). Similarly, dimorphic taxa exhibited a greater upper bound of differentiation than trimorphic taxa. Alongside this, polyphenisms within trimorphic species (with five overall ‘parts’) were differentiated more regularly than many monomorphic and dimorphic species (with fewer parts), that is, their polyphenisms are distributed more regularly across morphospace. Singular or more synthetic indices of complexity would not capture this variation.

We can use such spaces to understand how morphology changes relative to the division of labour within a colony. Enhanced division of labour suggests that interdependent parts in a system, like different polyphenisms within a species, will differentiate to perform fewer tasks more efficiently. Thus, we might see an interaction between the number of parts in a system and the degree of differentiation or the regularity of their arrangement. This could manifest in two ways: colonies with more worker polymorphisms might occupy a larger morphospace, with new polymorphisms expanding into new areas to fulfil novel functions. Alternatively, all colonies might occupy a similar morphospace volume, as they must perform a similar set of functions. Colonies with fewer polymorphisms would fill this space with greater distances between each ‘part’, while those with more polymorphisms would populate morphospace with intermediate worker polymorphisms. Polymorphic workers could perform existing tasks with greater morphological specificity than monomorphic workers, who must perform multiple tasks.

Our results are equivocal with respect to both hypotheses. While the lower bound of differentiation was similar across species with three, four and five parts, the upper bound decreased as the number of parts increased. Similarly, maximum irregularity levels decreased with more polyphenisms, with dimorphic and trimorphic species being more evenly distributed across morphospace than monomorphic species. Notably, many of the most regularly differentiated species were dimorphic.

These findings suggest that in terms of head shape, monomorphic species (male, queen, worker) tend to exhibit higher differentiation than dimorphic and trimorphic species. Although dimorphic and trimorphic workers show lower head shape differentiation, they expand more broadly into ecomorphospace through size differentiation (which we did not quantify). It is surprising that many monomorphic species occupy an area of complexity space characterized by low differentiation, low part number and low irregularity. This partially supports the hypothesis that parts tend to differentiate to encompass a similar morphospace area. Species with fewer parts may do this through shape differentiation, while those with more parts may achieve it through size differentiation. Monomorphic species probably appear most irregular because they have fewer polyphenisms to cover the colony’s full functional repertoire, leading to greater and less regular morphofunctional gaps between males, queens and workers. Conversely, in dimorphic and trimorphic species, there is a more regular distribution relative to the number of parts, as worker–worker phenotypes are more similar than worker–queen and worker–male phenotypes, resulting in a lower CV.

When sorted by colony size, our complexity space shows a strong correlation between the number of worker polymorphisms and colony size. Monomorphic species (with three parts) almost exclusively live in smaller colonies, while dimorphic and trimorphic species mostly inhabit colonies with more than 1000 individuals. Other indices of complexity, such as the degree and irregularity of part differentiation, do not correlate with colony size. Notably, however, five species living in colonies with fewer than 100 individuals occupy an area of complexity space characterized by low part number, medium differentiation and high irregularity. This underscores problems inherent in combining indices of social and morphological complexity into single metrics.

## 12. Conclusions and applications

Studies of biological complexity are a rapidly burgeoning area of macroevolutionary research. There are no universal indices of biological complexity, which is a multivariate concept and therefore most appropriately quantified relative to a multidimensional complexity space. Here, we conceive of three-dimensional spaces defined by orthogonal axes of part number, part differentiation and the regularity of part arrangement [126,127]. We note the potential to expand the dimensionality further provided new indices satisfy an information-based framework. Complexity spaces can be applied to systems at various levels of biological organization. Here, we offer contrasting examples of the proportions of somites within the thoraxes of trilobites (serial homologues within organisms) and the two-dimensional shapes of the heads of attine ants of different polyphenisms and worker polymorphisms within a colony or ‘superorganism’. The first example uses just two linear measurements (pleural and axial lobe width) while the second utilizes much more complex data in the guise of elliptical Fourier decompositions of shape [128,129]. In both cases, we extract indices on all three of our dimensions and demonstrate that systems (organisms and colonies) can vary on these axes independently.

Moreover, our results highlight the necessity of a delineated, multivariate approach for quantifying configurational complexity and its potential for both answering and precipitating new questions that will lead to a greater understanding of how and why complexity changes through time. Namely, the surprising early segment number and differentiation in the trilobites, and the functional significance of intermediate worker forms in the ants, merit further inquiry in separate research and should utilize our multidimensional approach. Many putative drivers of complexity make predictions about change in particular indices. With complexity spaces, these indices can be considered independently and relative to one another and can give greater resolution to hypotheses regarding the functional and non-functional significance of trends in complexity through time and in different spatial and temporal contexts.

## Figures and Tables

**Figure 7.**
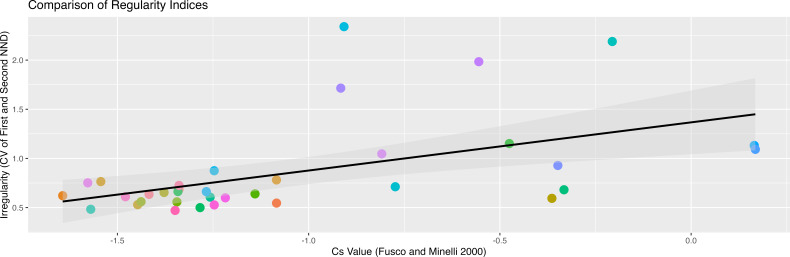
Comparison of our regularity index, CV of first and second NND with Fusco & Minelli’s [24] 'Cs' regularity index, calculated using every measurement from our trilobite dataset. Each point is coloured by taxon, consistent with the colouring scheme in figures 2 and 4.

**Figure 8.**
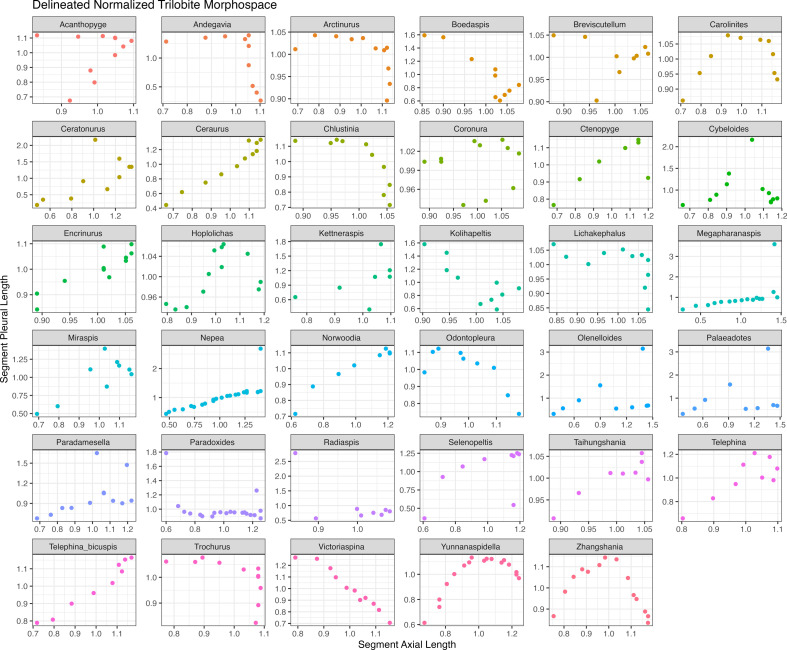
Independent morphospace distributions of thoracic segments for each trilobite taxon, as shown in figure 2. Axes of segment distributions are scaled to their respective ranges, not the actual ranges of their distributions in morphospace.

**Figure 9.**
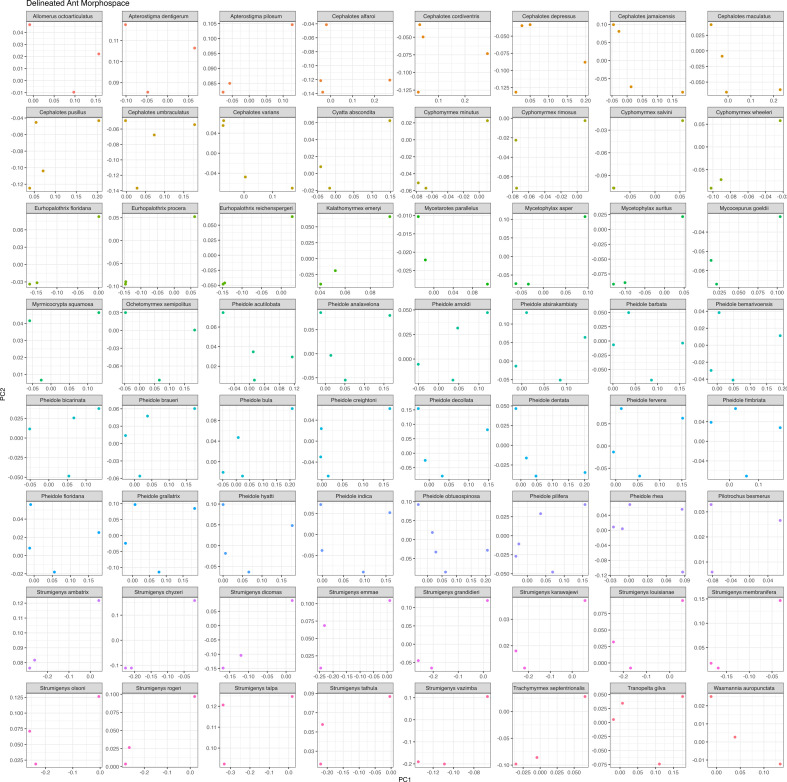
Independent morphospace distributions of polyphenisms for each ant taxon (PC1 and PC2), as shown in figure 5. Axes of polyphenism distributions are scaled to their respective ranges, not the actual ranges of their distributions in morphospace.

## Data Availability

All images and code used for analysis are available through electronic supplementary material hosted on Zenodo [130], as are the morphological, ecological and occurrence data. All data manipulation and analyses were performed using custom R scripts with existing R packages referenced in the main text. This code is available to replicate analyses and figure plotting.

## Appendix A. Comparing indices of regularity

We compare our index of regularity, CV of first- and second-order NNDs, with a pre-existing index established by Fusco & Minelli [24]. Their index ‘Cs’ measures morphological variation between adjacent serial homologues in a sequence,

$$Cs=log\left(\sum_{i=2}^{n-1}\left|2X_{i}-X_{i-1}-X_{i+1}\right|N\right),$$

where $X_{i}$ is a single morphometric measurement taken from the *i*th element of a series of homologues (e.g. the *i*th vertebrae in a spine), and *N* is the total number of elements (e.g. total number of vertebrae).

Their index cannot be applied to our ant dataset, given that there is no clear procession of ‘parts’ (polyphenisms) in an ant species, but can be applied to our trilobite dataset. To accommodate for the multiple morphometric values taken for each trilobite body segment (namely the mediolateral lengths of each thoracic pleural and axial lobe), we modified their equation to

$$Cs=log\left(\sum_{i=i}^{n}\left|E_{i}\right|N\right),$$

where $E_{i}$ is the Euclidean distance between all *S* values for the *i*th element of a series, and *N* is the total number of elements in the series. *S* values of the *i*th element are calculated as

$$S_{i}=2X_{i}-X_{i-1}-X_{i+1},$$

where $X_{i}$ is a single morphometric measurement taken from the *i*th element of a series. This method represents a geometric average of morphometric distances between each homologous part in a series and can be extended to accommodate datasets with any number of morphometric measurements for each homologue in a series.

Our comparison suggests that our index of regularity (CV of first and second NND) generally produces similar results to the index devised by Fusco & Minelli [24], albeit with the potential for outliers at higher Cs and CV values. While we believe that CV is an appropriate measurement, we acknowledge that more sophisticated metrics could be developed. Nonetheless, CV has wide utility and makes few assumptions about the composition of parts in a system and their development (figure 7).

## Appendix B. Complexity indices: trilobites

**Table 1.** Complexity indices for trilobite taxa. Regularity is measured as the CV of first- and second-order NNDs.

genus	part number	differentiation	regularity	first occurrence (Ma)
*Acanthopyge*	11	0.027	0.684	433
*Andegavia*	10	0.209	0.545	419
*Arctinurus*	11	0.024	0.621	449
*Boedaspis*	10	0.128	0.781	468
*Breviscutellum*	9	0.005	0.764	416
*Carolinites*	10	0.031	0.529	488
*Ceratonurus*	10	0.465	0.593	423
*Ceraurus*	11	0.104	0.653	471
*Chlustinia*	10	0.028	0.559	460
*Coronura*	11	0.005	0.558	402
*Ctenopyge*	7	0.046	0.639	541
*Cybeloides*	12	0.184	1.151	471
*Encrinurus*	11	0.009	0.662	478
*Hoplolichas*	12	0.017	0.499	470
*Kettneraspis*	7	0.164	0.681	460
*Kolihapeltis*	10	0.1	0.606	419
*Lichakephalus*	11	0.01	0.482	485
*Megapharanaspis*	17	0.533	2.189	516
*Miraspis*	9	0.102	0.711	460
*Nepea*	23	0.263	2.340	504
*Norwoodia*	8	0.064	0.874	541
*Odontopleura*	9	0.027	0.662	460
*Olenelloides*	9	0.89	1.131	519
*Palaeadotes*	9	0.879	1.092	505
*Paradamesella*	12	0.103	0.928	505
*Paradoxides*	20	0.081	1.715	513
*Radiaspis*	8	0.522	1.984	449
*Selenopeltis*	9	0.148	1.046	485
*Taihungshania*	8	0.005	0.751	485
*Telephina*	9	0.035	0.599	471
*Telephina bicuspis*	9	0.042	0.526	466
*Trochurus*	11	0.015	0.610	449
*Victoriaspina*	11	0.041	0.470	485
*Yunnanaspidella*	17	0.055	0.725	524
*Zhangshania*	14	0.031	0.634	516

## Appendix C. Complexity indices: ants

**Table 2.** Complexity indices for trilobite taxa. Regularity is measured as the CV of first- and second-order NNDs.

species	part number	differentiation	regularity
*Allomerus octoarticulatus*	3	0.01	0.274
*Apterostigma dentigerum*	3	0.014	0.382
*Apterostigma pilosum*	3	0.015	0.477
*Cephalotes alfaroi*	4	0.047	0.383
*Cephalotes cordiventris*	4	0.023	0.744
*Cephalotes depressus*	4	0.019	0.471
*Cephalotes jamaicensis*	4	0.029	0.581
*Cephalotes maculatus*	4	0.029	0.516
*Cephalotes pusillus*	4	0.01	0.505
*Cephalotes umbraculatus*	4	0.021	0.380
*Cephalotes varians*	4	0.024	0.591
*Cyatta abscondita*	3	0.013	0.517
*Cyphomyrmex minutus*	3	0.009	0.455
*Cyphomyrmex rimosus*	3	0.008	0.214
*Cyphomyrmex salvini*	3	0.012	0.445
*Cyphomyrmex wheeleri*	3	0.011	0.456
*Eurhopalothrix floridana*	3	0.017	0.504
*Eurhopalothrix procera*	3	0.026	0.525
*Eurhopalothrix reichenspergeri*	3	0.019	0.486
*Kalathomyrmex emeryi*	3	0.005	0.429
*Mycetarotes parallelus*	3	0.009	0.539
*Mycetophylax asper*	3	0.018	0.562
*Mycetophylax auritus*	3	0.014	0.278
*Mycocepurus goeldii*	3	0.007	0.496
*Myrmicocrypta squamosa*	3	0.011	0.371
*Ochetomyrmex semipolitus*	3	0.01	0.226
*Pheidole acutilobata*	4	0.006	0.333
*Pheidole analavelona*	4	0.012	0.299
*Pheidole arnoldi*	4	0.007	0.312
*Pheidole atsirakambiaty*	4	0.013	0.094
*Pheidole barbata*	4	0.009	0.079
*Pheidole bemarivoensis*	4	0.014	0.335
*Pheidole bicarinata*	4	0.007	0.216
*Pheidole braueri*	4	0.012	0.290
*Pheidole bula*	4	0.018	0.381
*Pheidole creightoni*	4	0.011	0.439
*Pheidole decollata*	4	0.017	0.289
*Pheidole dentata*	4	0.014	0.408
*Pheidole fervens*	4	0.011	0.180
*Pheidole fimbriata*	4	0.012	0.084
*Pheidole floridana*	4	0.011	0.328
*Pheidole grallatrix*	4	0.02	0.188
*Pheidole hyatti*	4	0.011	0.190
*Pheidole indica*	4	0.012	0.159
*Pheidole obtusospinosa*	5	0.013	0.391
*Pheidole pilifera*	5	0.009	0.415
*Pheidole rhea*	5	0.01	0.462
*Pilotrochus besmerus*	3	0.011	0.444
*Strumigenys ambatrix*	3	0.033	0.621
*Strumigenys chyzeri*	3	0.043	0.476
*Strumigenys dicomas*	3	0.023	0.446
*Strumigenys emmae*	3	0.03	0.341
*Strumigenys grandidieri*	3	0.03	0.528
*Strumigenys karawajewi*	3	0.028	0.560
*Strumigenys louisianae*	3	0.022	0.451
*Strumigenys membranifera*	3	0.016	0.623
*Strumigenys olsoni*	3	0.025	0.490
*Strumigenys rogeri*	3	0.03	0.556
*Strumigenys talpa*	3	0.044	0.627
*Strumigenys tathula*	3	0.018	0.533
*Strumigenys vazimba*	3	0.04	0.574
*Trachymyrmex septentrionalis*	3	0.008	0.324
*Tranopelta gilva*	4	0.014	0.427
*Wasmannia auropunctata*	3	0.004	0.348

## References

[1] Rebout N, Lone JC, De Marco A, Cozzolino R, Lemasson A, Thierry B. 2021 Measuring complexity in organisms and organizations. R. Soc. Open Sci. **8**, 200895. (10.1098/rsos.200895) 33959307 PMC8074971

[2] Parrott L. 2010 Measuring ecological complexity. Ecol. Indic. **10**, 1069–1076. (10.1016/j.ecolind.2010.03.014)

[3] Riva F, Graco-Roza C, Daskalova GN, Hudgins EJ, Lewthwaite JMM, Newman EA, Ryo M, Mammola S. 2023 Toward a cohesive understanding of ecological complexity. Sci. Adv. **9**, eabq4207. (10.1126/sciadv.abq4207) 37343095 PMC10284553

[4] Grizzi F, Chiriva-Internati M. 2005 The complexity of anatomical systems. Theor. Biol. Med. Model. **2**, 26. (10.1186/1742-4682-2-26) 16029490 PMC1180857

[5] West SA, Fisher RM, Gardner A, Kiers ET. 2015 Major evolutionary transitions in individuality. Proc. Natl Acad. Sci. USA **112**, 10112–10119. (10.1073/pnas.1421402112) 25964342 PMC4547252

[6] Böttcher T. 2018 From molecules to life: quantifying the complexity of chemical and biological systems in the universe. J. Mol. Evol. **86**, 1–10. (10.1007/s00239-017-9824-6) 29260254 PMC5794832

[7] Szathmáry E, Smith JM. 1995 The major transitions in evolution. Oxford, UK: Oxford University Press.

[8] McShea DW. 2001 The hierarchical structure of organisms: a scale and documentation of a trend in the maximum. Paleobiology **27**, 405–423. (10.1666/0094-8373(2001)0272.0.co;2)

[9] Changizi MA. 2001 Universal scaling laws for hierarchical complexity in languages, organisms, behaviors and other combinatorial systems. J. Theor. Biol. **211**, 277–295. (10.1006/jtbi.2001.2346) 11444957

[10] Corning PA. 1998 'The synergism hypothesis': on the concept of synergy and its role in the evolution of complex systems. J. Soc. Evol. Syst. **21**, 133–172. (10.1016/s1061-7361(00)80003-x)

[11] Keenan JP, McShea DW. 2023 Synergies among behaviors drive the discovery of productive interactions. Biol. Theory **18**, 43–62. (10.1007/s13752-022-00420-2)

[12] Artime O, De Domenico M. 2022 From the origin of life to pandemics: emergent phenomena in complex systems. Phil. Trans. R. Soc. A **380**, 20200410. (10.1098/rsta.2020.0410) 35599559 PMC9125231

[13] Szathmáry E, Smith JM. 1995 The major evolutionary transitions. Nature **374**, 227–232. (10.1038/374227a0) 7885442

[14] Noble D. 2012 A theory of biological relativity: no privileged level of causation. Interface Focus **2**, 55–64. (10.1098/rsfs.2011.0067) 23386960 PMC3262309

[15] Mazzocchi F. 2012 Complexity and the reductionism-holism debate in systems biology. Wiley Interdiscip. Rev. Syst. Biol. Med. **4**, 413–427. (10.1002/wsbm.1181) 22761024

[16] Gardiner JD, Behnsen J, Brassey CA. 2018 Alpha shapes: determining 3D shape complexity across morphologically diverse structures. BMC Evol. Biol. **18**. (10.1186/s12862-018-1305-z) PMC6282314 30518326

[17] Reichert J, Backes AR, Schubert P, Wilke T. 2017 The power of 3D fractal dimensions for comparative shape and structural complexity analyses of irregularly shaped organisms. Methods Ecol. Evol. **8**, 1650–1658. (10.1111/2041-210x.12829)

[18] Adamowicz SJ, Purvis A, Wills MA. 2008 Increasing morphological complexity in multiple parallel lineages of the Crustacea. Proc. Natl Acad. Sci. USA **105**, 4786–4791. (10.1073/pnas.0709378105) 18347335 PMC2290764

[19] Brinkworth A, Green E, Li Y, Oyston J, Ruta M, Wills MA. 2023 Bird clades with less complex appendicular skeletons tend to have higher species richness. Nat. Commun. **14**, 5817. (10.1038/s41467-023-41415-2) 37726273 PMC10509246

[20] Cisne JL. 1974 Evolution of the world fauna of aquatic free-living arthropods. Evolution **28**, 337–366. (10.1111/j.1558-5646.1974.tb00757.x) 28564844

[21] Li Y, Brinkworth A, Green E, Oyston J, Wills M, Ruta M. 2023 Divergent vertebral formulae shape the evolution of axial complexity in mammals. Nat. Ecol. Evol. **7**, 367–381. (10.1038/s41559-023-01982-5) 36878987 PMC9998275

[22] McShea DW. 1992 A metric for the study of evolutionary trends in the complexity of serial structures. Biol. J. Linn. Soc. **45**, 39–55. (10.1111/j.1095-8312.1992.tb00630.x)

[23] Jones KE, Angielczyk KD, Pierce SE. 2019 Stepwise shifts underlie evolutionary trends in morphological complexity of the mammalian vertebral column. Nat. Commun. **10**, 5071. (10.1038/s41467-019-13026-3) 31699978 PMC6838112

[24] Fusco G, Minelli A . 1999 Measuring morphological complexity of segmented animals: centipedes as model systems. J. Evol. Biol. **13**, 38–46. (10.1046/j.1420-9101.2000.00139.x)

[25] McShea DW. 1993 Evolutionary change in the morphological complexity of the mammalian vertebral column. Evolution **47**, 730–740. (10.1111/j.1558-5646.1993.tb01229.x) 28567892

[26] McShea DW. 2005 The evolution of complexity without natural selection, a possible large-scale trend of the fourth kind. Paleobiology **31**, 146–156. (10.1666/0094-8373(2005)031[0146:teocwn]2.0.co;2)

[27] Gould SJ. 2002 The structure of evolutionary theory, 5th edn. Cambridge, MA: Harvard University Press. (10.4159/9780674417922)

[28] Valentine JW. 2004 On the origin of phyla. Chicago, IL: University of Chicago Press. See https://books.google.co.uk/books?id=DMBkmHm5fe4C.

[29] Adami C. 2002 What is complexity? BioEssays **24**, 1085–1094. (10.1002/bies.10192) 12447974

[30] Molnar JL, Diogo R. 2021 Evolution, homology, and development of tetrapod limb muscles. Diversity **13**, 393. (10.3390/d13080393)

[31] Owen R. 2007 On the nature of limbs: a discourse. Chicago, IL: University of Chicago Press. (10.7208/chicago/9780226641959.001.0001)

[32] Herculano-Houzel S. 2011 Not all brains are made the same: new views on brain scaling in evolution. Brain Behav. Evol. **78**, 22–36. (10.1159/000327318) 21691045

[33] Herculano-Houzel S, Collins CE, Wong P, Kaas JH. 2007 Cellular scaling rules for primate brains. Proc. Natl Acad. Sci. USA **104**, 3562–3567. (10.1073/pnas.0611396104) 17360682 PMC1805542

[34] Williams RW, Herrup K. 1988 The control of neuron number. Annu. Rev. Neurosci. **11**, 423–453. (10.1146/annurev.ne.11.030188.002231) 3284447

[35] Herculano-Houzel S, Catania K, Manger PR, Kaas JH. 2015 Mammalian brains are made of these: a dataset of the numbers and densities of neuronal and nonneuronal cells in the brain of Glires, Primates, Scandentia, Eulipotyphlans, Afrotherians and Artiodactyls, and their relationship with body mass. Brain Behav. Evol. 86, 145–163. (10.1159/000437413) 26418466

[36] Stark G, Pincheira-Donoso D. 2022 The evolution of brain size in ectothermic tetrapods: large brain mass trades-off with lifespan in reptiles. Evol. Biol. **49**, 180–188. (10.1007/s11692-022-09562-4)

[37] Wills MA, Briggs DEG, Fortey RA. 1998 Evolutionary correlates of arthropod tagmosis: scrambled legs. In Arthropod relationships, pp. 57–65. Dordrecht, The Netherlands: Springer. (10.1007/978-94-011-4904-4_6)

[38] McShea DW, Brandon RN. 2010 Biology’s first law: the tendency for diversity and complexity to increase in evolutionary systems. Chicago, IL: University of Chicago Press. (10.7208/chicago/9780226562278.001.0001)

[39] Stoltzfus A. 1999 On the possibility of constructive neutral evolution. J. Mol. Evol. **49**, 169–181. (10.1007/pl00006540) 10441669

[40] Cooper GA, West SA. 2018 Division of labour and the evolution of extreme specialization. Nat. Ecol. Evol. **2**, 1161–1167. (10.1038/s41559-018-0564-9) 29807994

[41] Ispolatov I, Ackermann M, Doebeli M. 2012 Division of labour and the evolution of multicellularity. Proc. R. Soc. B **279**, 1768–1776. (10.1098/rspb.2011.1999) PMC3297448 22158952

[42] Hopkins MJ, Gerber S. 2017 Morphological disparity. In Evolutionary developmental biology: a reference guide (eds L Nuno de la Rosa, G Müller), pp. 1–12. Cham, Switzerland: Springer. (10.1007/978-3-319-33038-9_132-1)

[43] Wills MA. 2001 Morphological disparity: a primer. In Topics in geobiology: fossils, phylogeny, and form (eds JM Adrain, GD Edgecombe, BS Lieberman), pp. 55–144. Boston, MA: Springer. (10.1007/978-1-4615-0571-6_4)

[44] Wills MA, Briggs DEG, Fortey RA. 1994 Disparity as an evolutionary index: a comparison of Cambrian and Recent arthropods. Paleobiology **20**, 93–130. (10.1017/s009483730001263x)

[45] Guillerme T *et al*. 2020 Disparities in the analysis of morphological disparity. Biol. Lett. **16**, 20200199. (10.1098/rsbl.2020.0199) 32603646 PMC7423048

[46] van den Ende C, Puttick MN, Urrutia AO, Wills MA. 2023 Why should we compare morphological and molecular disparity? Methods Ecol. Evol. **14**, 2390–2410. (10.1111/2041-210x.14166)

[47] Minelli A. 2016 Species diversity vs. morphological disparity in the light of evolutionary developmental biology: Table 1. Ann. Bot. **117**, 781–794. (10.1093/aob/mcv134) 26346718 PMC4845798

[48] Hughes M, Gerber S, Wills MA. 2013 Clades reach highest morphological disparity early in their evolution. Proc. Natl Acad. Sci. USA **110**, 13875–13879. (10.1073/pnas.1302642110) 23884651 PMC3752257

[49] Villalobos F, Arita HT. 2014 Morphological diversity at different spatial scales in a Neotropical bat assemblage. Oecologia **176**, 557–568. (10.1007/s00442-014-3039-y) 25159214

[50] Guillerme T, Puttick MN, Marcy AE, Weisbecker V. 2020 Shifting spaces: which disparity or dissimilarity measurement best summarize occupancy in multidimensional spaces? Ecol. Evol. **10**, 7261–7275. (10.1002/ece3.6452) 32760527 PMC7391566

[51] Puttick MN, Guillerme T, Wills MA. 2020 The complex effects of mass extinctions on morphological disparity. Evol. Int. J. Org. Evol. **74**, 2207–2220. (10.1111/evo.14078) 32776526

[52] Hinegardner R, Engelberg J. 1983 Biological complexity. J. Theor. Biol. **104**, 7–20. (10.1016/0022-5193(83)90398-3)

[53] Kolmogorov AN. 1968 Three approaches to the quantitative definition of information. Int. J. Comput. Math. **2**, 157–168. (10.1080/00207166808803030)

[54] Papentin F. 1980 On order and complexity. I. General considerations. J. Theor. Biol. **87**, 421–456. (10.1016/0022-5193(80)90230-1)

[55] Sharma A, Czégel D, Lachmann M, Kempes CP, Walker SI, Cronin L. 2023 Assembly theory explains and quantifies selection and evolution. Nature **622**, 321–328. (10.1038/s41586-023-06600-9) 37794189 PMC10567559

[56] Stone J. 2015 Information theory: a tutorial introduction, 2nd edn. New York, NY: Sebtel Press. (10.13140/2.1.1633.8240)

[57] Lesne A. 2014 Shannon entropy: a rigorous notion at the crossroads between probability, information theory, dynamical systems and statistical physics. Math. Struct. Comp. Sci. **24**, e240311. (10.1017/S0960129512000783)

[58] Rioul O. 2021 This is IT: a primer on Shannon’s entropy and information. In Information theory (eds B Duplantier, V Rivasseau), pp. 49–86. Cham, Switzerland: Birkhäuser. (10.1007/978-3-030-81480-9_2)

[59] Shannon CE. 1948 A mathematical theory of communication. Bell Syst. Tech. J. **27**, 379–423. (10.1002/j.1538-7305.1948.tb01338.x)

[60] Baddeley A, Rubak E, Turner R. 2015 Spatial point patterns: methods and applications with R. New York, NY: Chapman and Hall/CRC. (10.1201/b19708)

[61] Dry M, Preiss K, Wagemans J. 2012 Clustering, randomness, and regularity: spatial distributions and human performance on the traveling salesperson problem and minimum spanning tree problem. J. Probl. Solving **4**. (10.7771/1932-6246.1117)

[62] Lev O, Edgecombe GD, Chipman AD. 2022 Serial homology and segment identity in the arthropod head. Integr. Org. Biol. **4**, obac015. (10.1093/iob/obac015) 35620450 PMC9128542

[63] Zrzavý J, Štys P. 1997 The basic body plan of arthropods: insights from evolutionary morphology and developmental biology. J. Evol. Biol. **10**, 353–367. (10.1046/j.1420-9101.1997.10030353.x)

[64] Clark E. 2021 Time and space in segmentation. Interface Focus **11**, 20200049. (10.1098/rsfs.2020.0049) 34055302 PMC8086912

[65] Pueyo JI, Lanfear R, Couso JP. 2008 Ancestral notch-mediated segmentation revealed in the cockroach Periplaneta americana. Proc. Natl Acad. Sci. USA **105**, 16614–16619. (10.1073/pnas.0804093105) 18927236 PMC2568983

[66] Zrzavy J, Stys P. 1995 Evolution of metamerism in Arthropoda: developmental and morphological perspectives. Q. Rev. Biol. **70**, 279–295. (10.1086/419072)

[67] Boxshall GA. 2004 The evolution of arthropod limbs. Biol. Rev. Camb. Philos. Soc. **79**, 253–300. (10.1017/s1464793103006274) 15191225

[68] Fusco G, Minelli A. 2013 Arthropod segmentation and tagmosis. In Arthropod biology and evolution: molecules, development, morphology (eds A Minelli, G Boxshall, G Fusco), pp. 197–221. Berlin, Germany: Springer. (10.1007/978-3-642-36160-9_9)

[69] Hopkins MJ, To R. 2022 Long-term clade-wide shifts in trilobite segment number and allocation during the Palaeozoic. Proc. R. Soc. B **289**. (10.1098/rspb.2022.1765) PMC9768642 36541173

[70] Hughes NC. 2003 Trilobite tagmosis and body patterning from morphological and developmental perspectives. Integr. Comp. Biol. **43**, 185–206. (10.1093/icb/43.1.185) 21680423

[71] Holmes JD. 2023 Contrasting patterns of disparity suggest differing constraints on the evolution of trilobite cephalic structures during the Cambrian ‘explosion’. Palaeontology **66**, e12647. (10.1111/pala.12647)

[72] Hopkins MJ. 2014 The environmental structure of trilobite morphological disparity. Paleobiology **40**, 352–373. (10.1666/13049)

[73] Foote M. 1989 Perimeter-based Fourier analysis: a new morphometric method applied to the trilobite cranidium. J. Paleontol. **63**, 880–885. (10.1017/s0022336000036556)

[74] Halliday A. 2022 Trilobite cephalon morphospace through time. MPhil thesis, University of Manchester, Manchester, UK.

[75] Gerber S. 2019 Use and misuse of discrete character data for morphospace and disparity analyses. Palaeontology **62**, 305–319. (10.1111/pala.12407)

[76] Pettie S, Ramachandran V. 2002 An optimal minimum spanning tree algorithm. J. ACM **49**, 16–34. (10.1145/505241.505243)

[77] Schneider CA, Rasband WS, Eliceiri KW. 2012 NIH Image to ImageJ: 25 years of image analysis. Nat. Methods **9**, 671–675. (10.1038/nmeth.2089) 22930834 PMC5554542

[78] Guillerme T. 2018 dispRity: A modular R package for measuring disparity. Methods Ecol. Evol. **9**, 1755–1763. (10.1111/2041-210x.13022)

[79] R.Core Team. 2021 *R: A language and environment for statistical computing*. Vienna, Austria: R Foundation for Statistical Computing. See https://www.R-project.org/.

[80] McNamara KJ. 1986 The role of heterochrony in the evolution of Cambrian trilobites. Biol. Rev. **61**, 121–156. (10.1111/j.1469-185x.1986.tb00464.x)

[81] Beecher CE. 1897 Outline of a natural classification of the trilobites. Am. J. Sci. **s4-3**, 89–106. (10.2475/ajs.s4-3.14.89)

[82] Hupé P. 1953 Classification des trilobites. Issy-les-Moulineaux, France: Masson. See https://books.google.co.uk/books?id=X6QAGwAACAAJ.

[83] Stubblefield CJ. 1959 Evolution in trilobites. Q.J.G.S. **115**, 145–162. (10.1144/GSL.JGS.1959.115.01.08)

[84] Laibl L, Saleh F, Pérez-Peris F. 2023 Drifting with trilobites: the invasion of early post-embryonic trilobite stages to the pelagic realm. Palaeogeogr. Palaeoclimatol. Palaeoecol. **613**, 111403. (10.1016/j.palaeo.2023.111403)

[85] Laibl L. 2017 Patterns in palaeontology: the development of trilobites. Palaeontol. Online **7**, 1–9.

[86] Edgecombe GD, Chatterton BDE. 1987 Heterochrony in the Silurian radiation of encrinurine trilobites. Lethaia **20**, 337–351. (10.1111/j.1502-3931.1987.tb00793.x)

[87] Fortey RA, Owens RM. 1999 Feeding habits in trilobites. Palaeontology **42**, 429–465. (10.1111/1475-4983.00080)

[88] Whittington HB. 1981 Paedomorphosis and cryptogenesis in trilobites. Geol. Mag. **118**, 591–602. (10.1017/s0016756800033823)

[89] Vrba ES, Gould SJ. 1986 The hierarchical expansion of sorting and selection: sorting and selection cannot be equated. Paleobiology **12**, 217–228. (10.1017/s0094837300013671)

[90] McKinney ML, McNamara KJ. 1991 Heterochrony. In Heterochrony, pp. 1–12. Boston, MA: Springer US. (10.1007/978-1-4757-0773-1_1)

[91] Hughes NC. 2003 Trilobite body patterning and the evolution of arthropod tagmosis. BioEssays **25**, 386–395. (10.1002/bies.10270) 12655645

[92] Hughes NC. 2007 Strength in numbers: high phenotypic variance in early Cambrian trilobites and its evolutionary implications. BioEssays **29**, 1081–1084. (10.1002/bies.20674) 17935151

[93] Webster M. 2007 A Cambrian peak in morphological variation within trilobite species. Science **317**, 499–502. (10.1126/science.1142964) 17656721

[94] Erwin DH. 2015 Novelty and innovation in the history of life. Curr. Biol. **25**, R930–40. (10.1016/j.cub.2015.08.019) 26439356

[95] Gould SJ. 1989 Wonderful Life: The burgess shale and the nature of history. New York, NY: W.W. Norton. See https://books.google.co.uk/books?id=4T6fQgAACAAJ.

[96] Marshall CR. 2006 Explaining the Cambrian 'explosion' of animals. Annu. Rev. Earth Planet. Sci. **34**, 355–384. (10.1146/annurev.earth.33.031504.103001)

[97] Webster M, Zelditch ML. 2011 Evolutionary lability of integration in Cambrian ptychoparioid trilobites. Evol. Biol. **38**, 144–162. (10.1007/s11692-011-9110-2)

[98] Saunders PT, Ho MW. 1976 On the increase in complexity in evolution. J. Theor. Biol. **63**, 375–384. (10.1016/0022-5193(76)90040-0) 1011851

[99] Queller DC, Strassmann JE. 2009 Beyond society: the evolution of organismality. Phil. Trans. R. Soc. B **364**, 3143–3155. (10.1098/rstb.2009.0095) 19805423 PMC2781869

[100] Wilson DS, Sober E. 1989 Reviving the superorganism. J. Theor. Biol. **136**, 337–356. (10.1016/s0022-5193(89)80169-9) 2811397

[101] Gardner A, Alpedrinha J, West SA. 2012 Haplodiploidy and the evolution of eusociality: split sex ratios. Am. Nat. **179**, 240–256. (10.1086/663683) 22218313

[102] Trivers RL, Hare H. 1976 Haploidploidy and the evolution of the social insect. Science **191**, 249–263. (10.1126/science.1108197) 1108197

[103] Hölldobler B, Wilson EO. 2009 The super organism: the beauty, elegance, and strangeness of insect societies. New York, NY: W. W. Norton & Company.

[104] Oster GF, Wilson EO. 1978 Caste and ecology in the social insects. Monogr. Popul. Biol. **12**, 1–352. 740003

[105] Ravary F, Lecoutey E, Kaminski G, Châline N, Jaisson P. 2007 Individual experience alone can generate lasting division of labor in ants. Curr. Biol. **17**, 1308–1312. (10.1016/j.cub.2007.06.047) 17629482

[106] Anderson C, McShea DW. 2001 Individual versus social complexity, with particular reference to ant colonies. Biol. Rev. Camb. Philos. Soc. **76**, 211–237. (10.1017/s1464793101005656)

[107] Holley JAC, Moreau CS, Laird JG, Suarez AV. 2016 Subcaste-specific evolution of head size in the ant genus Pheidole. Biol. J. Linn. Soc. **118**, 472–485. (10.1111/bij.12769)

[108] Wilson EO. 1985 The sociogenesis of insect colonies. Science **228**, 1489–1495. (10.1126/science.228.4707.1489) 17831241

[109] Robinson G. 1992 Regulation of division of labor in insect societies. Annu. Rev. Entomol. **37**, 637–665. (10.1146/annurev.ento.37.1.637) 1539941

[110] Ferguson-Gow H, Sumner S, Bourke AFG, Jones KE. 2014 Colony size predicts division of labour in attine ants. Proc. R. Soc. B **281**, 20141411. (10.1098/rspb.2014.1411) PMC4173680 25165765

[111] Rodriguez-Serrano E, Inostroza-Michael O, Avaria-Llautureo J, Hernandez CE. 2012 Colony size evolution and the origin of eusociality in corbiculate bees (Hymenoptera: Apinae). PloS One **7**, e40838. (10.1371/journal.pone.0040838) 22808274 PMC3396608

[112] Fisher B, Fong J. 2024 AntWeb. San Francisco, CA: California Academy of Sciences. See https://www.gbif.org/dataset/13b70480-bd69-11dd-b15f-b8a03c50a862.

[113] Csősz S, Báthori F, Rádai Z, Herczeg G, Fisher BL. 2023 Comparing ant morphology measurements from microscope and online AntWeb.org 2D z‐stacked images. Ecol. Evol. **13**, e9897. (10.1002/ece3.9897) 36950369 PMC10025076

[114] Mehdiabadi NJ, Schultz TR. 2010 Natural history and phylogeny of the fungus-farming ants (Hymenoptera: Formicidae: Myrmicinae: Attini). Myrmecol. NEWS **13**, 37–55.

[115] Trible W, Kronauer DJC. 2017 Caste development and evolution in ants: it’s all about size. J. Exp. Biol. (eds JD Levine, DJC Kronauer, MH Dickinson), **220**, 53–62. (10.1242/jeb.145292) 28057828

[116] Casadei‐Ferreira A, Feitosa RM, Pie MR. 2022 Size and shape in the evolution of the worker head in Pheidole ants (Hymenoptera: Formicidae). J. Zool. **317**, 270–282. (10.1111/jzo.12978)

[117] Pie MR, Tschá MK. 2013 Size and shape in the evolution of ant worker morphology. PeerJ **1**, e205. (10.7717/peerj.205) 24255818 PMC3828632

[118] Wilson EO. 2003 Pheidole in the new world. a dominant, hyperdiverse ant genus. Cambridge, MA: Harvard University Press.

[119] Bonhomme V, Picq S, Gaucherel C, Claude J. 2014 Momocs: outline analysis using R. J. Stat. Softw **56**, 13. (10.18637/jss.v056.i13)

[120] Bourke. 1999 Colony size, social complexity and reproductive conflict in social insects. J. Evol. Biol. **12**, 245–257. (10.1046/j.1420-9101.1999.00028.x)

[121] La Richelière F *et al*. 2022 Warm and arid regions of the world are hotspots of superorganism complexity. Proc. R. Soc. B **289**. (10.1098/rspb.2021.1899) PMC8832517 35135345

[122] Rajakumar R *et al*. 2018 Social regulation of a rudimentary organ generates complex worker-caste systems in ants. Nature **562**, 574–577. (10.1038/s41586-018-0613-1) 30305737

[123] Molet M, Maicher V, Peeters C. 2014 Bigger helpers in the ant Cataglyphis bombycina: increased worker polymorphism or novel soldier caste? PloS One **9**, e84929. (10.1371/journal.pone.0084929) 24404196 PMC3880325

[124] Powell S. 2016 A comparative perspective on the ecology of morphological diversification in complex societies: nesting ecology and soldier evolution in the turtle ants. Behav. Ecol. Sociobiol. **70**, 1075–1085. (10.1007/s00265-016-2080-8)

[125] Holland JG, Bloch G. 2020 The complexity of social complexity: a quantitative multidimensional approach for studies of social organization. Am. Nat. **196**, 525–540. (10.1086/710957) 33064587

[126] McShea DW. 1996 Perspective metazoan complexity and evolution: is there a trend? Evolution **50**, 477–492. (10.1111/j.1558-5646.1996.tb03861.x) 28568940

[127] Mitteroecker P, Huttegger SM. 2009 The concept of morphospaces in evolutionary and developmental biology: mathematics and metaphors. Biol. Theory **4**, 54–67. (10.1162/biot.2009.4.1.54)

[128] Bookstein FL. 1992 Morphometric tools for landmark data: geometry and biology. Cambridge, UK: Cambridge University Press. (10.1017/CBO9780511573064)

[129] James Rohlf F, Marcus LF. 1993 A revolution morphometrics. Trends Ecol. Evol. **8**, 129–132. (10.1016/0169-5347(93)90024-J) 21236128

[130] Rock T. 2024 ESM: Quantifying the configurational complexity of biological systems in multivariate complexity space. Zenodo. See 10.5281/zenodo.14501061.39875092

